# Effect of *Allium sativum* consumption for breast feeding among postnatal Indian mothers

**DOI:** 10.6026/97320630019698

**Published:** 2023-06-30

**Authors:** Mahalakshmi B, Jignasaben Patani, Gnanadesigan Ekambaram, Padmavathi P

**Affiliations:** Department of Pediatric nursing, Nootan College of Nursing, Sankalchand Patel University, Visnagar-384315, Gujarat, India; Department of Community Medicine, Nootan Medical College and Research Centre, Sankalchand Patel University, Visnagar-384315, Gujarat, India; Department of Physiology, Nootan Medical College and Research Centre, Sankalchand Patel University, Visnagar-384315, Gujarat, India; Department of Biochemistry, ACS Medical College and Hospital, DR MGR Education and Research institute University, Chennai-600077,Tamil Nadu, India

**Keywords:** Effect, *Allium sativum* consumption, breastfeeding, post natal mother

## Abstract

Breast milk is a unique form of nutrition for babies since it provides all of the essential nutrients for growth and development. Breastfeeding may also play a role in decreasing postpartum depression, and reduced the risk of breast and ovarian cancer in future. Garlic has been used as a galactogogue in India since many years. The study's major goal was to determine the effect of garlic consumption in promotion of breast feeding among the postnatal mothers in Nootan General Hospital Visnagar, India. The study employed a pre-experimental one-group pre and post-test research design, with the sample drawn via non-probability convenience sampling. The baseline preform and the 12-point modified adequacy of breast feeding checklist were used to collect data. The mean pre-test score was 3.33, and the mean post-test score was 8.33 in this study's data analysis. 5.47 was the average difference. The post-test mean was lower than the pre-test mean, indicating that garlic preparation consumption promotes breastfeeding among postnatal mothers. The pre-test stress score had a standard deviation of 2.56, while the post-test stress score had a standard deviation of 2.57.

## Background:

Breastfeeding is important for the health of new born and mothers. Breast milk is a unique form of nutrition for babies since it provides all of the essential nutrients for growth and development. Antibodies and lymphocytes from the mother are found in breast milk, which assist the infant fight illnesses. The nutritional content of breast milk is specially formulated to optimize growth and reduce the risk of obesity, sudden infant death syndrome (SIDS). Breastfeeding may also play a role in decreasing postpartum depression, and reduced the risk of breast and ovarian cancer in future [1]. Infants who are breastfed exclusively for six months had lower rates of gastrointestinal infection morbidity than infants who stop breastfeeding after three or four months, and there have been no growth impairments in infants shown[2].The immune function of breast milk is customized because the mother comes into contact with pathogens that colonies the new born through her touching and caring for the baby, and her body produces the proper antibodies and immune cells as a result [3].To promote maximum health and development, the World Health Organization (WHO) recommends that new borns be exclusively breastfeeding for the first six months of life, followed by supplementary foods while maintaining breastfeeding for up to two years or longer. However, worldwide, only about 40% of infants under the age of six months are exclusively breastfed [2]. Many mothers are concerned that they will not be able to provide enough milk for their children. A woman's milk production may be hampered by a physical or hormonal problem that makes it difficult to build or sustain. Insufficient milk supply is described as a situation in which a woman has or believes she has an insufficient amount of breast milk to satiate and/or maintain the infant's weight increase [4]. Insufficient milk production makes moms feel failed at breastfeeding and mothering, causing them to stop breastfeeding, too soon. In one national research on feeding behaviors, approximately half of mothers said they stopped nursing due to a lack of milk production. Infrequent feeding or poor nursing technique can result in a low milk production. However, a lack of confidence in nursing or a lack of understanding of the typical physiology of lactation can lead to the perception of a low milk supply, despite the fact that [5] garlic (*Allium sativum*) has been used to lower cholesterol and blood pressure. It has no specific indications for use during lactation in western countries. Garlic has been used as a galactogogue in India and Turkey,[6,7, 8] although no scientific data could be located on its use alone as a galactogogue. Garlic's odor is transmitted to breast milk, which may increase infant sucking time acutely and might enhance the breastfed infant's food choices in the long term. Vitamins, minerals, and amino acids are all found in garlic. It is also contains sulphur compounds, which are responsible for the majority of its health benefits as well as its pungent odour. Garlic has been used to cure infection, edema, and digestive issues for millennia. Garlic consumption by the mother a few hours before nursing encourages the baby to suck the breast because it alters the flavour of the mother's milk. As a result, garlic is safe to use while breastfeeding. Eating garlic or taking garlic supplements may support lactation in some women. Although no research exists to show its effectiveness, some people in India use garlic. Despite claims that Garlic is a highly effective functional food in improving the breast milk production, seldom there are studies to prove they are effective in improving prolactin levels and explaining their impact on breast milk production. Therefore, it is of interest to study the effect of *Allium sativum* (Garlic) consumption in relation to effective breast feeding.

## Materials and Methods:

The Study took a pre-experimental research approach. The research was carried out in Nootan General Hospital Visnagar. The study's research design was a pre-experimental, one-group and pre and post-test design. A non-probability sampling strategy was used to choose the hospital and the sample from it. Study was approved by institutional ethics committee, and all subjects gave their informed consent after the study methods and goal were explained in local language (Gujarati). The experiments followed the amended Helsinki Declaration of 1975 that was revised in 2013.The baseline preform and the 12-point modified adequacy of breast feeding checklist were used to collect data. Six postnatal mothers who met the sampling criteria participated in a pilot study. A total of 60 postpartum mothers were included in the study, with breastfeeding promotion ranging from inadequate to moderate. After estimating the pre-test using an observational checklist, the post-natal mother was given hot and sour soup twice a day for a week. A postnatal mother's post-test was performed on the seventh day. To make data analysis easier, the data was tabulated in a methodical manner. Descriptive and inferential statistics were used to analyze the acquired data.

## Statistical analysis:

SPSS (Statistical Package for Social Sciences) was used to conduct the statistical analysis (version 17.0) of Descriptive and inferential statistics. The Student's t-test is used to examine the results, which are given as mean standard deviation (SD). A statistically significant P-value of 0.05 was used. The Chi-square test was used to determine the relationship between breastfeeding promotion and demographic variables.

## Results:

**Figure 1:**
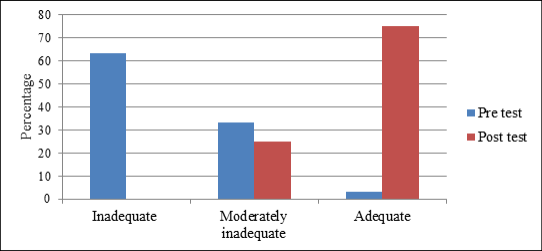
Percentage distribution of pre-test and post-test of breastfeeding adequacy among post natal mothers exposed to intake of garlic preparation

The demographic factors of the present study show (Table 1), the majority of the samples were between the ages of 22 and 25 (41.66 percent). The majority of the samples had a secondary health education as their educational level (50 percent). The majority of the participants were from nuclear households (61.66 percent). The vast majority of the samples were Hindu religion (90 percent). The majority of the samples were homemakers (61.66 percent). Rural area has the highest percentage of the sample (42 percent). The percentage of married people was the highest (60 percent). The majority of the samples were from caesarean sections in the lower portion (68.33 percent). The proportion of samples with two children is the highest (55 percent). According to the data, 63.33 percent of postpartum women did not support breastfeeding effectively. According to postnatal data, 75% of postnatal mothers had adequately promoted breastfeeding (Table 2 and Figure 1). Garlic consumption has an effect. The mean post-test score (8.802.35) is higher than the mean pre-test score (3.332.56) in postnatal mothers (Table 3). At the 0.05 level of significance, the calculated value (12.024) is bigger than the table value (1.67). As a result, the null hypothesis was disproved. After eating garlic, the mean post-test promotion of breastfeeding score (8.802.35) in postnatal mothers was 8.802.35. As a result, the null hypothesis was disproved and the hypothesis was confirmed. The Chi-square test (Table 4) was used to determine the relationship between breastfeeding promotion and demographic variables. All other characteristics had no statistical significant relationship with postnatal mother's posttest breastfeeding adequacy. At the 0.05 level of significance, age and employment status were shown to be substantially linked with pre-test level. As a result, just a portion of the research hypothesis was accepted.

## Discussion:

*Allium sativum* (garlic) is one of the most researched medicinal plants. The chemistry and biological effects of garlic and garlic products has been the subject of more than three thousand research publications published between 1960 and 2007. The cardiovascular, anti-microbial, and anti-cancer properties of garlic are the main subjects of these investigations. Garlic has been used to lower cholesterol and blood pressure. It has no specific indications for use during lactation in western countries. Garlic has been used as a galactogogue in India since many years. There was no proper study to investigate the effects of garlic on breast feeding. In the present study garlic intake shows predominant action on breast feeding. Garlic intake promotes breast feeding effectively among post natal mothers. It is in agreement with ACOG Committee Opinion in which they studied with garlic capsules [9]. Each capsule contains 1.5 g of garlic extract. Garlic-naive infants whose mothers ingested garlic capsules spent more time (33 vs 27 minutes) attached to the nipple during the time period of 1.5 to 3 hours after garlic ingestion. Infants who received garlic in the milk for the first time spent 30% more time nursing than after Placebo (which is given as control). It shows similar impact in the present study. Only difference is mode of intake of garlic. Scheffler *et al*. shows that six nursing mothers of 22 to 51 weeks postpartum period donated 3 milk samples via breast pump after eating 3 grams of raw garlic 1 before ingesting the garlic and 2 afterwards at 2 to 3 hour intervals [10]. Scheffler *et al*. also shows that after consuming garlic, metabolites produced from it were monitored for up to 5.2 hours [10]. A panel of sensory experts evaluated scents in milk samples, and high resolution gas chromatography-olfactometry identified numerous known metabolites in the milk. They also found that only allyl methyl sulphide, allyl methyl sulfoxide, and allyl methyl sulfone were detected in breast milk out of 13 potential garlic-derived metabolites. Only the allyl methyl sulphide metabolite smelled like garlic; the other two metabolites had no smell. The measured metabolite levels reached their maximum levels 2 to 3 hours after maternal garlic consumption.

A study from Mennella *et al.*. reported that after taking 1.5 g of garlic in capsule form once daily, mothers retrieved 20 mL of breast milk hourly for the following 4 hours [11]. 11 men and women who were blinded to the consumption of maternal products judged the smell of garlic in each sample. The strongest garlic smell was noticed two hours after maternal garlic consumption. The stench was still detectable in some mother's breast milk even after three hours. Another study done by Qin *et al*. who investigated the effects of *Allium sativum* (garlic) among mothers ingesting roasted and cooked (boiled) garlic [12]. According to his study, after consuming roasted and boiled garlic by postpartum mothers shows increased milk secretion and also found allyl methyl sulphide in milk samples. They also found that chemical compounds such as allyl methyl sulfoxide and allyl methyl sulfone peak levels from 1 to 4 hours after ingestion of roasted and cooked garlic, although levels were lower after cooked garlic. In our present study we didn't go through any chemical analysis after intake of garlic enriched food as like Qin *et al*. and Scheffler *et al*. indeed it sis in agreement with our reports.

## Limitations of the present study:

There are a few limitations of the study. In the present study, we couldn't be able to do chemical analysis of effect of *Allium sativum* (garlic) after food. Hence, in feature, we would like to include chemical analysis of *Allium sativum* with large number of sample size.

## Conclusion:

Data shows that intake of garlic preparation was helpful in promoting breastfeeding among experimental groups. Breastfeeding should be encouraged. More studies on the effects of *Allium sativum* are needed to be conducted in future with large population.

## Figures and Tables

**Table 1:** A frequency and percentage distribution of sample according to their demographic variables

**S.NO**	**Characteristics**	**Categories**	**Frequency(F)**	**Percentage (%)**
1	Age	>21year	10	16.66%
		21-25year	25	41.66%
		26-25year	21	35%
		31-35year	4	6.66%
2	Educational status	Primary	20	33.33%
		Secondary	30	50%
		Undergraduate	10	16.66%
		Post graduate	0	0%
3	Occupationstatus	Homemaker	37	61.66%
		Employed	16	26.66%
		Self-employed	7	11.66%
4	Religion	Hindu	54	90%
		Christian	0	0%
		Muslim	6	10%
		Others	0	0%
5	Residence	Urban	18	30%
		Rural	42	70%
6	Maritalstatus	Married	60	100%
		Unmarried	0	0%
7	Type of family	Nuclear family	37	61.66%
		Jointfamily	23	38.33%
8	Mode of delivery	Normal delivery	17	28.33%
		LSCS	41	68.33%
		Forceps delivery	2	3.33%
9	Number of child	One	21	35%
		Two	33	55%
		More than two	6	10%
10	Knowledge about Alternative therapies To promote breast feeding	Yes	5	8.33%
		No	55	91.66%

**Table 2:** Frequency distribution of pre-test and post-test of breastfeeding adequacy among post natal mothers exposed to intake of garlic preparation

	**Pre - test**	**Post-test**
Breast feeding adequacy in postnatal mother	F	F
Inadequate( 0-4 )	38	0
*Moderately* inadequate( 5-8 )	20	15
Adequate( 9-12 )	2	45

**Table 3:** Mean, S.D, Mean difference and 't' value of pre-test and post-test Breast milk adequacy scores of effectiveness of intake garlic preparation. DF=n-1(60-1) = 59

**Para meter**	**Mean**	**S.D.**	**M.D.**	**t'value**	**Table 't' Value**	**Level of Significance 0.05**
Pre-test	3.33	2.5 6	5.47	12.024	1.67	S
Post-Test	8.8	2.33	

**Table 4:** Chi-square analysis of relationship between post-test breastfeeding adequacy and selected demographic characteristics among postnatal mothers

	**Variables**	**Category**	**Frequency**	**Breast milk adequacy**		**d.f.**	**Table value**	**Chi-Square test**	**Significant >0.05 %**
				**Mild Adequate (5-8)**	**Adequate (9-12)**				
1	Age	>21year	10	5	5	3	7.59	8.57	S
		21-25year	25	17	8				
		26-25year	21	20	1				
		31-35year	4	3	1				
2	Educational status	Primary	20	15	5	2	5.84	0.17	NS
		Secondary	30	23	7				
		Undergraduate	10	7	3				
		Postgraduate	0	0	0				
3	Occupational status	Homemaker	37	30	7	2	5.84	7.95	S
		Employed	16	15	1				
		Self-employed	7	3	4				
4	Religion	Hindu	54	43	11	1	3.82	0.29	NS
		Christian	0	0	0				
		Muslim	6	2	4				
		Others	0	0	0				
5	Residence	Urban	18	14	4	1	3.82	0.1	NS
		Rural	42	31	11				
6	Marital status	Married	60	45	15	1	3.82	0.18	NS
		Unmarried	0	0	0				
7	Type Of family	Nuclear family	37	30	7	1	3.82	1.9	NS
		Joint family	23	15	8				
8	Type of delivery	Normal delivery	17	12	5	2	5.84	1.46	NS
		LSCS	41	32	9				
		Forceps delivery	2	1	1				
9	Number of children	One	21	16	5	2	5.84	0.26	NS
		Two	33	26	7				
		More than two	6	3	3				
10	Knowledge about alternative therapies promotion of breast milk	Yes	5	1	4	1	3.82	0.7	NS
		No	55	44	11				
